# Measurable Residual Disease Assessment in B-Lymphoblastic Leukemia Using 5- and 10-Color Flow Cytometry: An Institutional Experience

**DOI:** 10.7759/cureus.101730

**Published:** 2026-01-17

**Authors:** Anand V Narayanan, Mohandoss Murugesan, Chandran Nair, Sangeetha K Nayanar, Jithin TK, Gopakumar KG

**Affiliations:** 1 Oncopathology, Malabar Cancer Centre, Kannur, IND; 2 Transfusion Medicine, Malabar Cancer Centre, Kannur, IND; 3 Clinical Hematology, Malabar Cancer Centre, Kannur, IND; 4 Pediatric Oncology, Malabar Cancer Centre, Kannur, IND

**Keywords:** b-all, flow cytometry, immunophenotype, mrd, post-induction

## Abstract

Background: Measurable residual disease (MRD) assessment by flow cytometry is an established prognostic tool in B-lymphoblastic leukemia (B-ALL). Advances in multicolor flow cytometry have enabled higher event acquisition and expanded immunophenotypic analysis; however, real-world data comparing 5-color and 10-color flow cytometry platforms remain limited.

Objectives: To evaluate MRD detection by 5-color and 10-color flow cytometry and to assess leukemia-associated immunophenotype (LAIP) expression patterns and concordance with post-induction morphology in patients with B-ALL.

Methods: This retrospective study included patients with B‑ALL who had diagnostic immunophenotyping and post‑induction MRD assessment between 2018 and 2024 at a single center. MRD was evaluated using predefined LAIPs on a 5‑color, lower‑event acquisition protocol and a 10‑color, higher‑event acquisition protocol. MRD was considered positive when the presence of ≥10 clustered aberrant events was identified, showing a coherent population with two or more abnormal antigen expression patterns. Clinical, morphological, and flow cytometric parameters were collected and analyzed.

Results: A total of 172 paired baseline and post‑induction MRD samples were studied. MRD was detected in 38 cases (22.1%), with similar overall positivity rates on the 5‑color (12, 23.5%) and 10‑color (26, 21.5%) platforms. CD58 overexpression emerged as the most reproducible LAIP on both assays, while the 10‑color panel allowed finer assessment of treatment‑related antigen shifts, including loss of CD10 and CD34 in a subset of MRD‑positive samples, and achieved lower limits of MRD detection. Notably, more than two‑thirds of MRD‑positive cases by flow cytometry were classified as negative on conventional morphology.

Conclusion: In this cohort, 5‑color flow cytometry provided practical MRD assessment in B‑ALL, whereas 10‑color flow cytometry offered greater immunophenotypic detail and improved recognition of low‑level residual disease. These findings support the routine use of flow cytometry‑based MRD evaluation alongside morphology for post‑induction response assessment and risk‑adapted management in B‑ALL.

## Introduction

Measurable (minimal) residual disease (MRD) monitoring by flow cytometry has become a cornerstone of risk stratification and treatment response assessment in acute lymphoblastic leukemia (ALL) [1-4]. Over the past two decades, multiple clinical trials have shown that MRD status at the end of induction is a strong predictor of subsequent relapse and survival and often outperforms traditional clinical, cytogenetic, and morphologic parameters. In most contemporary B‑ALL treatment protocols, achieving an MRD level below 0.01% (10⁻⁴) at defined time points is regarded as a marker of good early response and is used to tailor therapy intensity [1,2].

Flow cytometry-based MRD detection relies on the identification of aberrant immunophenotypic patterns expressed by leukemic blasts that differ from normal B-cell maturation. These aberrancies include asynchronous antigen expression, over- or under-expression of lineage markers, and cross-lineage antigen expression, collectively referred to as leukemia-associated immunophenotypes (LAIPs) [4,5].

At diagnosis, one or more LAIPs are defined and subsequently tracked during follow-up to detect residual leukemic cells. In addition, many laboratories incorporate a different-from-normal (DfN) approach, particularly when immunophenotypic shifts occur during therapy [6].

The sensitivity and reliability of flow cytometric MRD assessment are influenced by several technical factors, including the number of fluorochromes used, antibody panel design, total events acquired, and analytical expertise [4-7]. Traditionally, MRD assays were performed using 4- or 5-color flow cytometry platforms, which allowed the evaluation of limited antigen combinations and typically acquired up to 500,000 total events per sample [1,4,7,8]. While these approaches have proven clinically useful and remain widely practiced, especially in resource-limited settings, their sensitivity may be constrained in cases with low-level disease or complex immunophenotypic patterns.

The introduction of higher-parameter flow cytometers has enabled the use of 8-10 color antibody panels, facilitating more comprehensive immunophenotypic characterization of leukemic blasts and improved discrimination from normal regenerating B-cell precursors (hematogones) [5,6,9]. Ten-color flow cytometry platforms permit the acquisition of higher total events (≥1,000,000), thereby enhancing analytical sensitivity and reducing the likelihood of false-negative MRD results, particularly at very low disease burdens [6,9].

Although higher-parameter flow cytometry is increasingly recommended for MRD assessment, many laboratories, particularly in resource-limited settings, continue to rely on 4- or 5-color platforms due to infrastructural and cost constraints. Most published studies evaluate multicolor MRD assays in highly standardized research environments, with limited data reflecting real-world institutional transitions from low-color, low-event to higher-color, high-event flow cytometry. This study addresses this gap by providing a longitudinal, real-world comparison of 5-color and 10-color flow cytometry for MRD assessment in B-ALL within routine clinical practice.

At our institution, flow cytometric MRD assessment in B-ALL was performed using a 5-color platform during the period from 2018 to 2020, followed by a transition to a 10-color flow cytometry system thereafter. Both methodologies are considered acceptable and are in routine clinical use; however, real-world data comparing LAIP expression patterns and MRD detection between low-event (5-color) and high-event (10-color) assays remain limited, particularly from resource-constrained settings.

The present study was undertaken to evaluate whether differences exist in the detection of MRD and the pattern of LAIP expression when assessed using 5-color versus 10-color flow cytometry in patients with B-ALL. The primary objective was to compare MRD detection rates between low-event and high-event flow cytometric assays, and the secondary objective was to assess the LAIP expression patterns and potential incremental value of higher-color panels in routine clinical practice.

## Materials and methods

Study design

This was a retrospective descriptive study involving review of flow cytometry (FCM) records of patients diagnosed with B-lymphoblastic leukemia (B-ALL) who underwent post-induction measurable residual disease (MRD) assessment. The study period extended from January 2018 to December 2024. The study was approved by the institutional review board and expedited review by the institutional ethics committee vide no: 1616/IRB-SRC/13/MCC/17-09-2022/3.

Study population

All patients with a confirmed diagnosis of B-ALL who had both baseline diagnostic immunophenotyping and post-induction (PI) MRD assessment by flow cytometry were eligible for inclusion. Patients with incomplete flow cytometry data and unavailable list mode data (LMD) files were excluded from analysis.

Flow cytometry data acquisition and analysis

Baseline diagnostic and post-induction MRD flow cytometry data were retrieved from institutional archives. During the study period, MRD analysis was performed using a 5-color flow cytometry platform until 2020 and a 10-color flow cytometry platform thereafter. 

Antibody panel composition

Baseline immunophenotyping and MRD assessment were performed using predefined leukemia-associated immunophenotype (LAIP)-based antibody panels.

5-color flow cytometry panel

The 5-color MRD assay was performed using a four-tube panel. Tube 1: CD20-FITC, CD10-PE, CD19-ECD, CD45-PC5. Tube 2: CD19-FITC, CD10-PE, CD58-PC5, CD45-ECD. Tube 3: CD19-FITC, CD10-PE, CD34-PC5, CD123-PC7, CD45-ECD. Tube 4: CD19-FITC, CD10-PE, CD34-PC5, CD38-PC7, CD45-ECD.

10-color flow cytometry panel

The 10-color MRD assay was performed using a two-tube panel. CD20-V450, CD45-V500c, CD86-BV605, CD58-FITC, CD66c/CD123-PE, CD34/CD79a-PerCP-Cy5.5, CD19-PE-Cy7, CD10-APC, CD200-APC-R700, CD38-APC-H7

For each case, FCM dot plots and list mode data files were reviewed. MRD analysis followed a sequential gating approach. Initially, nucleated cells were identified using forward scatter (FSC) and side scatter (SSC) properties, followed by exclusion of debris and doublets. CD45 versus SSC gating was used to identify the blast region (CD45 dim, low SSC). Within this region, B-lineage cells were selected using CD19 expression. Leukemia-associated immunophenotypes (LAIPs) were identified at diagnosis based on aberrant antigen expression patterns, including asynchronous expression, over- or under-expression of B-lineage markers, and cross-lineage antigen expression [6]. These predefined LAIPs were subsequently tracked during post-induction MRD evaluation.

The total number of events acquired per sample and the expression pattern of LAIP markers were documented for both baseline and MRD samples.

Operational definitions

Low-event MRD assay: acquisition of ≤500,000 total events, and high-event MRD assay: acquisition of ≥1,000,000 total events. MRD positivity: presence of a distinct cluster of ≥20 events showing aberrant immunophenotype with at least two concordant abnormal antigen expressions consistent with the diagnostic LAIP. MRD was quantified when a minimum of 40 clustered MRD events were identified, and results were expressed as a percentage of total nucleated events acquired [3].

Software and data management

Baseline and post-induction MRD list mode data files were analyzed using CXP software (Beckman Coulter) for 5-color flow cytometry data and BD FACSuite software (BD Biosciences) for 10-color flow cytometry data. Demographic and clinical data were extracted from electronic medical records. All study data were entered into Microsoft Excel and subsequently analyzed using IBM Corp. Released 2022. IBM SPSS Statistics for Windows, Version 29. Armonk, NY: IBM Corp.

Quality control

Daily instrument calibration and performance verification were performed using standardized fluorescent calibration beads according to manufacturer instructions and protocol.

Statistical analysis

Descriptive statistics were used to summarize demographic, clinical, and laboratory variables. Categorical variables were expressed as frequencies and percentages, while continuous variables were summarized as mean with standard deviation or median with range, as appropriate. The chi-square test was employed to assess the association between MRD results obtained from low-event and high-event assays. A two-sided p-value of <0.05 was considered statistically significant.

## Results

A total of 172 paired baseline and post-induction (PI) MRD samples from patients with B-lymphoblastic leukemia were included in the analysis. Leukemia-associated immunophenotypes (LAIPs) were identified in all cases at baseline and were subsequently reassessed during MRD evaluation. MRD positivity was defined and reported in accordance with EuroFlow recommendations [7].

The median age of the study population was 8 years (range: 1-59 years), with a predominantly pediatric cohort 109 (63.4%). There were 102 males (59.3%) and 70 females (40.7%).

The median blast percentage in baseline peripheral blood samples was 40% (range: 0-97%), while bone marrow samples showed a median blast percentage of 88% (range: 3-98%). The median total leukocyte count was 8,265 cells/µL (range: 390-328,000 cells/µL), and the median platelet count was 45 × 10³ cells/µL (range: 3-330 × 10³ cells/µL).

Flow cytometry platforms and event acquisition

Baseline immunophenotyping was performed using 5-color flow cytometry in 51 cases and 10-color flow cytometry in 113 cases. Post-induction MRD assessment was performed using 5-color flow cytometry in 51 cases and 10-color flow cytometry in 121 cases.

The median number of events acquired per sample was 500,000 for the 5-color platform and 2.25 million for the 10-color platform. On day eight, peripheral blood blast counts exceeding 1,000 cells/µL were noted in nine patients (5.2%). Post-induction bone marrow assessment demonstrated <5% blasts in 157 patients (92.4%), 5-24% blasts in nine patients, and >25% blasts in four patients; bone marrow data were unavailable for two patients. Post-induction bone marrow morphology was positive for residual disease in 13 patients (7.6%). Post-induction bone marrow assessment is routinely incorporated into ALL treatment protocols to enable early risk stratification and guide therapy intensification in poor early responders [2,4].

MRD detection and LAIP expression

Overall, MRD positivity was detected in 38 of 172 cases (22.1%). The MRD positivity rates were comparable between the two platforms, with 12 of 51 cases (23.5%) in the 5-color cohort and 26 of 121 cases (21.5%) in the 10-color cohort.

LAIP expression patterns on 5-color flow cytometry

**Figure 1:**
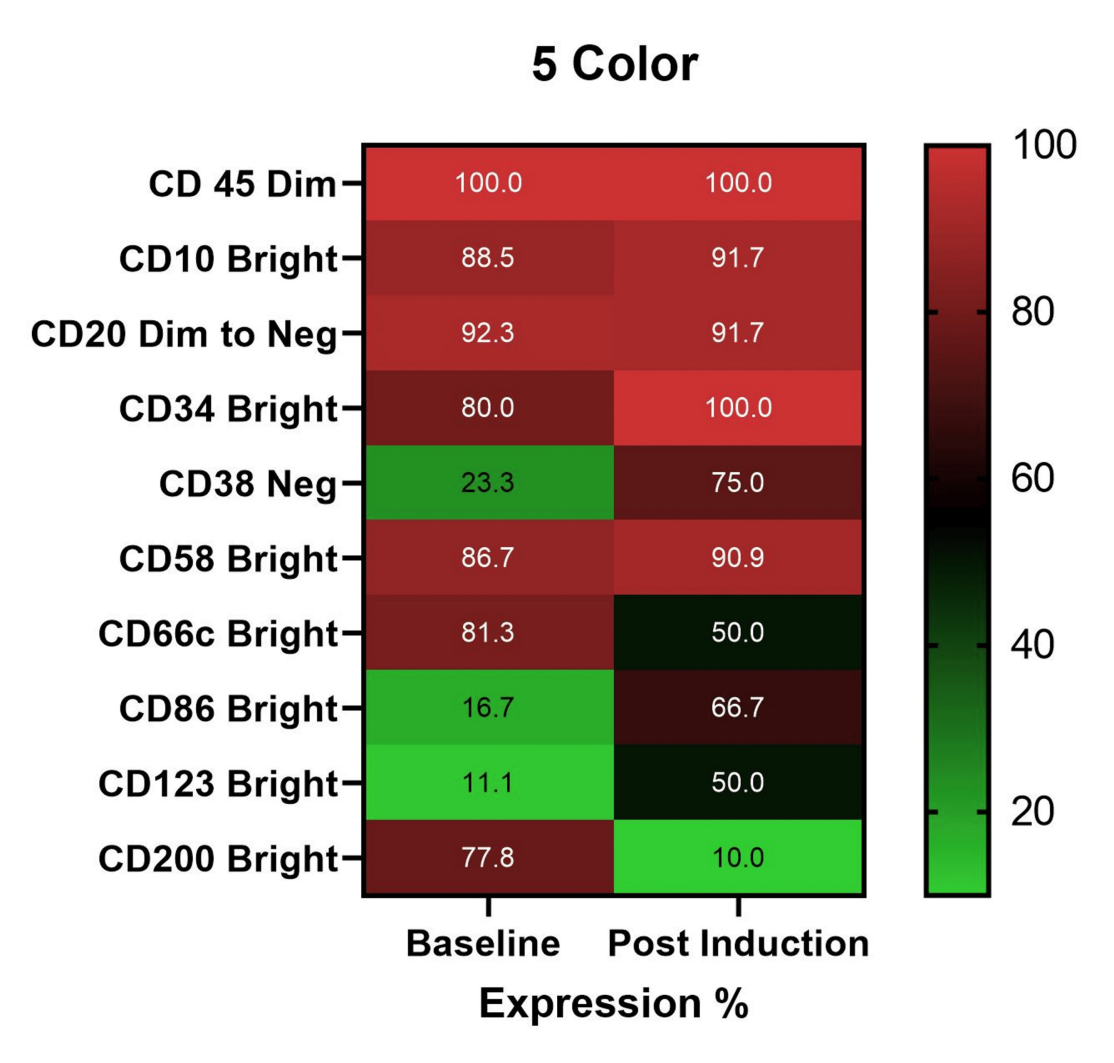
Expression of LAIP markers in 5-color equipment at baseline and post-induction LAIP: Leukemia-associated immunophenotype

As illustrated in Figure 1, LAIP markers showed high concordance between baseline and post-induction MRD samples in the 5-color platform. Core B-lineage and blast-associated markers, including CD45 (dim), CD10 (bright), CD20 (dim to negative), and CD34 (bright), were consistently expressed in the majority of cases at both time points (Table 1).

**Table 1:** Pattern of immunophenotype expression in B-ALL in baseline and post induction samples from a South Indian Cancer Centre ‡ Median (Range); ND: Not done, MRD: Measurable residual disease

Time Point	Platform	n	CD45 Dim	CD19 Bright†	CD10 Bright	CD20 Dim/Neg	CD34 Bright	CD38 Neg	CD58 Bright	CD66c Bright	CD86 Bright	CD123 Bright	CD200 Bright
Baseline	5-color	51	100	100	91.7	66.7	100	25.0	86.7	81.3	16.7	11.1	77.8
	10-color	121	100	100	69.2	53.8	46.2	4.0	73.1	38.5	19.2	ND	42.3
MRD-positive	5-color	12	100	2.7‡ (1.7–8.8)	91.7	91.7	91.7	25.0	83.3	50.0	66.7	50.0	10.0
	10-color	26	100	4.1‡ (2.4–13.7)	65.4	84.6	53.8	12.5	80.8	42.3	20.8	18.2	48.0

Among aberrant markers, CD58 overexpression was the most frequently observed LAIP, present in 33 (86.7%) of baseline samples and retained in 10 (90.9%) of MRD-positive post-induction samples. In contrast, CD38 negativity, CD86 overexpression, and CD123 overexpression were less frequent at baseline but showed relatively higher representation among MRD-positive cases, suggesting selective persistence of leukemic subclones with these aberrancies. CD200 overexpression, although common at baseline, was infrequently retained in MRD-positive samples.

LAIP expression patterns on 10-color flow cytometry

**Figure 2:**
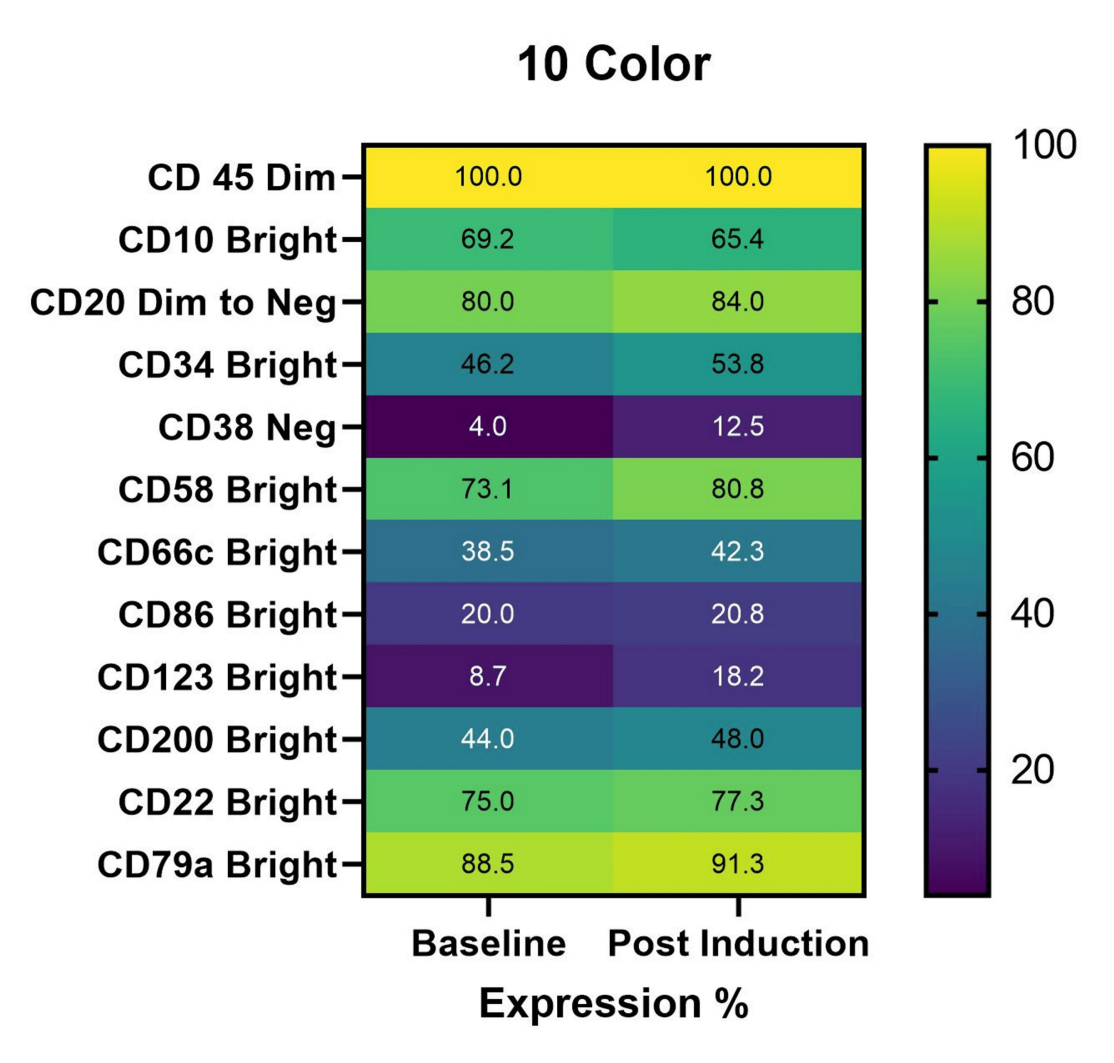
Expression of LAIP markers in 10-color equipment at baseline and post-induction LAIP: Leukemia-associated immunophenotype

The 10-color platform demonstrated greater heterogeneity in LAIP expression patterns (Figure 2). While CD45 (dim) expression was uniform across all samples, conventional B-lineage markers such as CD10, CD20, and CD34 showed variable expression, with partial loss observed in a subset of MRD-positive cases (Table 1).

Notably, aberrant markers, including CD38 dim to negativity, CD66c, CD86, CD123, and CD200, were overexpressed and more readily characterized in the 10-color assay, enabling improved delineation of leukemic populations from regenerating hematogones.

MRD burden and sensitivity

Among MRD-positive cases, the median proportion of CD19-positive gated cells was 2.7% (range: 1.7-8.8%) in the 5-color group and 4.1% (range: 2.4-13.7%) in the 10-color group. The lowest MRD levels detected were 0.015% with the 5-color assay and 0.012% with the 10-color assay, reflecting improved sensitivity associated with higher event acquisition.

Importantly, two MRD-positive cases detected using the 10-color platform demonstrated the absence of both CD10 and CD34, highlighting immunophenotypic shifts that may not be reliably identified using lower-color panels. This underscores the advantage of extended antibody combinations in detecting residual leukemic populations with altered antigen expression.

Post-induction bone marrow morphology and flow cytometry MRD results were available for 170 patients (Table 2). Flow cytometry identified MRD positivity in 38 cases, of which 12 cases were also positive by morphology, while 26 cases showed morphologically negative marrow despite MRD positivity on flow cytometry.

**Table 2:** Concordance between post-induction morphology and flow cytometry MRD MRD: Measurable residual disease

Post-Induction Flow Cytometry	Morphology (n=170)	
	Positive	Negative
MRD Positive	12	26
MRD Negative	1	131

Among the 132 cases that were MRD negative by flow cytometry, 131 cases were also morphology negative, and one case showed morphologic positivity in the absence of detectable MRD by flow cytometry. This discordance may reflect a known limitation of morphology, as regenerating hematogones can closely mimic leukemic blasts on light microscopy, particularly during early post-induction marrow regeneration.

## Discussion

Measurable residual disease (MRD) assessment has emerged as the most powerful prognostic indicator in B-lymphoblastic leukemia (B-ALL), guiding risk stratification and therapeutic decision-making across pediatric and adult treatment protocols [1-3]. In this institutional study, we evaluated MRD detection using 5-color and 10-color flow cytometry platforms and examined the concordance between post-induction morphology and flow cytometry-based MRD assessment. Our findings reaffirm the clinical utility of flow cytometry for MRD detection and highlight the incremental advantages of higher-color, high-event assays in routine practice.

In the present cohort, MRD positivity was detected in 38 (22.1%) of patients, a rate comparable to that reported in prior flow cytometry-based MRD studies at similar post-induction time points [2,4]. Importantly, MRD positivity rates were comparable between 5-color and 10-color platforms, suggesting that both methodologies remain clinically valid for MRD assessment. However, qualitative differences in immunophenotypic resolution and sensitivity were evident with the use of extended antibody panels and higher event acquisition in the 10-color platform.

A key observation of this study was the substantial discordance between post-induction morphology and flow cytometry MRD results. More than two-thirds of MRD-positive cases identified by flow cytometry were morphologically negative, underscoring the limited sensitivity of conventional morphology for detecting low-level residual disease. This finding is consistent with previous reports demonstrating that morphology typically detects leukemic blasts only when disease burden exceeds 5%, whereas flow cytometry can reliably identify residual disease at levels as low as 0.01% or below [1,5].

Analysis of leukemia-associated immunophenotypes (LAIPs) revealed that certain aberrancies, particularly CD58 overexpression, were consistently retained from diagnosis to post-induction MRD across both platforms. CD58 has been well described as a stable and highly discriminatory LAIP in B-ALL and is particularly useful in distinguishing leukemic blasts from regenerating hematogones during marrow recovery [6]. Conversely, markers such as CD10 and CD34 demonstrated variable expression, with partial or complete loss in a subset of MRD-positive cases, particularly those detected on the 10-color platform. Such immunophenotypic shifts during therapy have been well documented and represent a recognized challenge in MRD assessment when limited antibody panels are used [7,8].

The enhanced ability of 10-color flow cytometry to detect MRD in cases with altered or atypical immunophenotypes highlights the advantage of extended panels. The inclusion of additional markers such as CD66c, CD86, CD200, and CD22 allows improved characterization of aberrant antigen expression and reduces the risk of false-negative MRD results, particularly in cases with lineage infidelity or antigen modulation [9,10]. In our study, the identification of MRD-positive cases lacking CD10 and CD34 expression exclusively on the 10-color platform underscores the clinical relevance of this expanded immunophenotypic coverage.

Another important technical consideration is total event acquisition. The higher median event count achieved with the 10-color platform enabled detection of MRD at lower levels compared to the 5-color assay. High-event acquisition has been shown to improve assay sensitivity and confidence in MRD quantification, particularly when residual leukemic populations are rare [8]. While both platforms achieved detection limits consistent with accepted clinical thresholds, the marginally lower MRD levels detected with the 10-color assay in our cohort support its use where resources permit. The 10-color platform demonstrated a marginally lower detectable MRD threshold (0.012%) compared to the 5-color assay (0.015%), but the small absolute difference and limited number of MRD-positive cases precluded meaningful statistical comparison of analytical sensitivity.

Emerging single-tube 12-color flow cytometry panels have shown enhanced ability to distinguish hematogones from leukemic blasts and high concordance with molecular MRD assays, suggesting that further expansion of multicolor platforms may offer additional gains in MRD sensitivity and standardization beyond current 10-color approaches [11].

A key strength of this study is its relatively large real-world cohort reflecting routine clinical practice in a resource-constrained setting, as well as the longitudinal institutional transition from 5-color to 10-color flow cytometry for MRD assessment. This provided a practical opportunity to evaluate LAIP stability, antigen modulation, and morphology-MRD discordance across successive technological platforms, generating data that are directly applicable to laboratories undergoing similar upgrades. This study has certain limitations. Its retrospective nature and single-center design may limit generalizability. Molecular MRD methods such as PCR or next-generation sequencing were not available for comparison, and clinical outcome correlations were beyond the scope of the present analysis. Additionally, baseline and MRD assessments were performed on different platforms across time periods, which may introduce inherent variability. Nevertheless, this reflects real-world practice in many institutions transitioning from lower- to higher-parameter flow cytometry systems.

## Conclusions

Both 5-color and 10-color flow cytometry were effective for MRD assessment in B-ALL; however, 10-color flow cytometry provided superior immunophenotypic resolution and improved detection of antigen modulation and low-level residual disease. CD58 was the most consistent leukemia-associated immunophenotype across both platforms, while higher MRD detection in the 5-color group likely reflected technical limitations of lower event acquisition.
